# From 1D to 3D Graphitic Carbon Nitride (Melon): A Bottom-Up Route via Crystalline Microporous Templates

**DOI:** 10.1021/acs.inorgchem.1c02769

**Published:** 2021-12-02

**Authors:** Niklas Stegmann, Yitao Dai, Edward Nürenberg, Wolfgang Schmidt

**Affiliations:** Max-Planck-Institut für Kohlenforschung, Kaiser-Wilhelm-Platz 1, 45470 Mülheim a.d. Ruhr, Germany

## Abstract

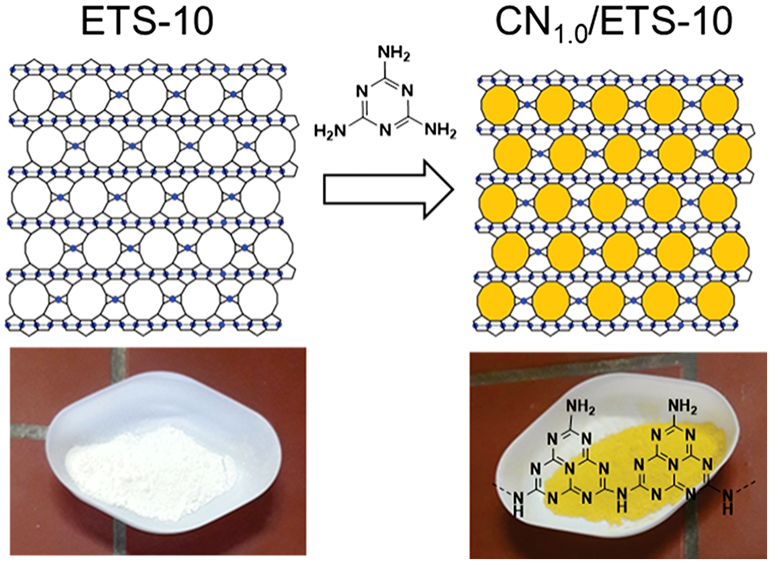

Herein, we present a novel bottom-up preparation route for heptazine-based polymers (melon), also known as graphitic carbon nitride. The growth characteristics of isolated 1D melon strings in microporous templates are presented and studied in detail. Removal of the microporous silicate template via etching is accompanied by the self-assembly of a 1D melon to stacked 3D structures. The advantages and limitations of the bottom-up approach are shown by using microporous templates with different pore sizes (ETS-10, ZSM-5, and zeolite Y). In accordance with the molecular size of the heptazine units (0.67 nm), a 1D melon can be deposited in ETS-10 with a pore width of about 0.78 nm, whereas its formation in the smaller 0.47 nm pores of ZSM-5 is sterically impeded. The self-assembly of isolated 1D melon to stacked 3D structures offers a novel experimental perspective to the controversial debate on the polymerization degree in 2D sheets of graphitic carbon nitride as micropore sizes below 1 nm confine the condensation degree of heptazine to isolated 1D strands at a molecular level. The growth characteristics and structural features were investigated by X-ray diffraction, N_2_ physisorption, scanning transmission electron microscopy/energy-dispersive X-ray analysis, ^13^C CP-NMR spectroscopy, and attenuated total reflection–infrared spectroscopy.

## Abstract 2

Isolated 1D melon strands grow in microporous silicate templates. Removal of the silicate template via etching goes along with the self-assembly of the 1D melon to stacked graphitic carbon nitride. The self-assembly of an isolated 1D melon offers a novel experimental perspective to the controversial debate on the polymerization of graphitic carbon nitride as pore sizes below 1 nm confine the condensation degree of heptazine to isolated 1D strands at a molecular level.

## Introduction

Heptazine-based allotropes of carbon nitride have emerged as a prominent class of materials known as graphitic carbon nitride (g-CN). Predominantly, structures comprising melon, a semi-condensed heptazine polymer, forming stacked 2D planes composed of 1D hydrogen-bonded polymer strands, are the subject of continuous research.^1,2^ Bulk g-CN can be facilely synthesized by thermally driven polycondensation reactions of nitrogen-rich precursors, such as cyanamide (H_2_N–CN) and its derivatives dicyanamide (H_2_N–CN)_2_ and melamine C_3_N_3_(NH_2_)_3_. Due to unique structural, chemical, and optical features, g-CN has found a broad range of applications as a functional material. Although facile precursor pyrolysis tends to form crystalline bulk g-CN, its applications as a functional material led to a wide range of sophisticated structure-directing approaches to optimize its performance. Structure-manipulated g-CNs result in the preparation of nano-sheets,^3^ nano-tubes,^4^ nano-rods,^5^ quantum dots,^6^ hollow spheres,^7^ thin films,^8^ porous frameworks,^9^ and various composites.^10−12^ These structure-directing approaches can be divided into “top-down” and “bottom-up” methods. Post-synthetic downsizing of bulk g-CN by thermal, chemical, or mechanochemical methods is characteristic for top-down strategies including the preparation of 2D sheets by thermal oxidation,^3^ chemical exfoliation,^13^ or ultrasound separation.^14^ Excessive down-sizing via multiple steps even results in 0D g-CN quantum dots.^6^ Bottom-up approaches primarily involve template-assisted synthesis routes divided into hard- and soft-templating methods. Depending on the template, g-CN structures with a defined porosity, particle size, and morphology are accessible via precursor polymerization. Soft-templating routes allowed the preparation of micro- and mesoporous g-CNs by using organic molecules as structure-directing agents, which are removed during the reaction process.^15,16^ In contrast, hard-templating methods employ inorganic materials as templates to direct the final structure of the target material, followed by template removal via etching. Several classes of hard templates have been used for the preparation of porous g-CNs, ranging from silica particles to porous foams up to crystalline materials with 3D pore systems.^9,17^ Particularly, templates with ordered mesoporous structures, such as KIT-6 or SBA-15, allow a precise control of the g-CN architectures and properties for their use as functional materials.^18,19^ Mesoporous materials are defined by a certain range of pore sizes (2–50 nm) and have been widely studied for the preparation of structured g-CN materials, whereas microporous templates (<2 nm) are not reported in the context of nanocasting routes. A first approach was made by Zhang et al. using microporous zeolite Y (cage size, 1.2 nm) to encapsulate g-CN by a two-step synthesis resulting in composites of zeolite Y and g-CNs with a confined polymerization degree.^20^ Nevertheless, reports on conventional nanocasting approaches using microporous templates, as known for a carbon replica of zeolites, do not exist up to now to the best of our knowledge.^21−24^ In this work, we investigate the sterical limits of hard-templating approaches for g-CNs using different microporous templates. In accordance with the molecular size of heptazine (0.67 nm), the growth characteristics of an isolated 1D melon are described in detail for templates with pore sizes below 1 nm. A suitable template for this approach is ETS-10, a microporous titanosilicate offering a 3D framework of interconnected channels with a pore size of about 0.78 nm.^25^ We show that the formation of heptazine units in pores smaller than the sizes of the heptazine units, as for example found in ZSM-5 (0.47 nm), is unfeasible for steric reasons and marks the molecular limit for templating g-CNs. Moreover, the experimental results offer a novel perspective on the formation and structure of g-CNs. The formation of layered 3D structures proceeds via the self-assembly of 1D melon after template removal. The condensation degree of heptazine units in the 2D plane of g-CNs has been the subject of numerous studies and is discussed controversially. Graphene-like sheets of fully condensed heptazine units (g-C_3_N_4_) have been postulated as an intralayer structure in g-CNs, but typical reaction conditions reported in the literature yielded semi-condensed melon structures.^2^ With respect to the presented approach, pore sizes below 1 nm inhibit the formation of graphene-like 2D sheets by confining the condensation degree of heptazine units to isolated 1D strands at a molecular level. The observed self-assembly behavior of a 1D melon to layered 3D g-CNs brings a new aspect to the structural debate from a novel experimental perspective.

## Experimental Section

### Template Preparation

A microporous ETS-10 template was synthesized under hydrothermal conditions from a gel with the molar composition of 5.4 SiO_2_:1.0 TiO_2_:1.6 Na_2_O:0.4 K_2_O:102 H_2_O using TiO_2_ (P 25, Degussa) and colloidal silica (40 wt % in H_2_O, Ludox AS 40, Sigma-Aldrich) as Ti and Si sources, respectively. In a typical preparation, 4.0 g of sodium hydroxide pellets (VWR Chemicals BDH, ≥97%) and 1.4334 g of potassium fluoride (Alfa Aesar, ≥99%) were dissolved in 41.66 g of deionized water in a polypropylene beaker. Under vigorous stirring, 2.46 g of P 25 was added to the solution, and subsequently, 25 g of Ludox AS 40 was admixed. The gel was stirred for 30 min and then transferred into 50 mL Teflon-lined stainless steel autoclaves to react under static conditions at 200 °C for 5 days. The resulting solid was washed with deionized water and dried at 90 °C. The detailed characterization of ETS-10 template is presented in the Supporting Information.

### Composite Formation

The preparation of the CN/ETS-10 composites was performed from mixtures of pristine ETS-10 and melamine (Aldrich, ≥99%). In a typical experiment, ETS-10 (1.0 g) was mixed with melamine (0.1–1.0 g) by shaking in a sealed vial. The mixture then was placed in a covered alumina crucible and heated in a furnace to 500 °C for 2 h under static air with a heating rate of 5 °C/min. A series of CN/ETS-10 composites were prepared with melamine/template weight ratios (*M*/*T* ratios) between 0.1 and 1.0, denoted as CN_(0.1–1.0)_/ETS-10.

For comparison, activated zeolite H-ZSM-5 (Süd-Chemie, Si/Al ≈ 13) and zeolite H–Y (Alfa Aesar, Si/Al ≈ 6) were used as alternative templates for comparison by mixing 1.0 g of the respective template with 1.0 g of melamine. The products are denoted as CN/ZSM-5 and CN/Y, respectively.

As a reference material, bulk g-CN was prepared under similar conditions by thermal polycondensation of melamine at 550 °C for 4 h using a heating rate of 5 °C/min but without adding any template.

### Template Removal

The templates were removed from the composites by treatment in 30 mL hydrofluoric acid solution (10 wt %, Alfa Aesar) at room temperature for 2 h under continuous shaking, followed by several centrifugation and washing steps with deionized water until pH 6–7 was achieved in the washing water. Template-free samples were denoted as R-CN_(0.1–1.0)_/ETS-10, with respect to their initial precursor ratios, R-CN/ZSM-5, and R-CN/Y, respectively.

## Results and Discussion

**Figure 1:**
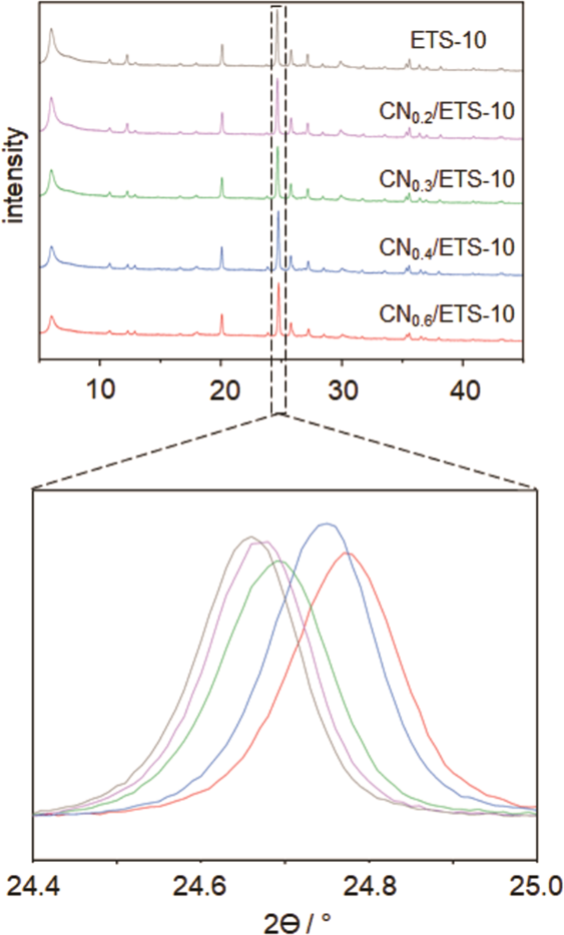
XRD patterns of pristine ETS10 (black) and CN_0.2–0.6_/ETS-10 composites [precursor ratio: 0.2 (purple), 0.3 (green), 0.4 (blue), and 0.6 (red)] with an increasing shift of the main reflection with an increasing *M*/*T* ratio.

The growth characteristics of carbon nitride polymers in microporous templates were investigated for CN/template composites with increasing *M*/*T* ratios as described in the Experimental Section. Figure 1 shows the X-ray diffraction (XRD) patterns of pristine microporous ETS-10 and CN/ETS-10 composites synthesized with increasing *M*/*T* ratios (0.2–0.6).

**Figure 2:**
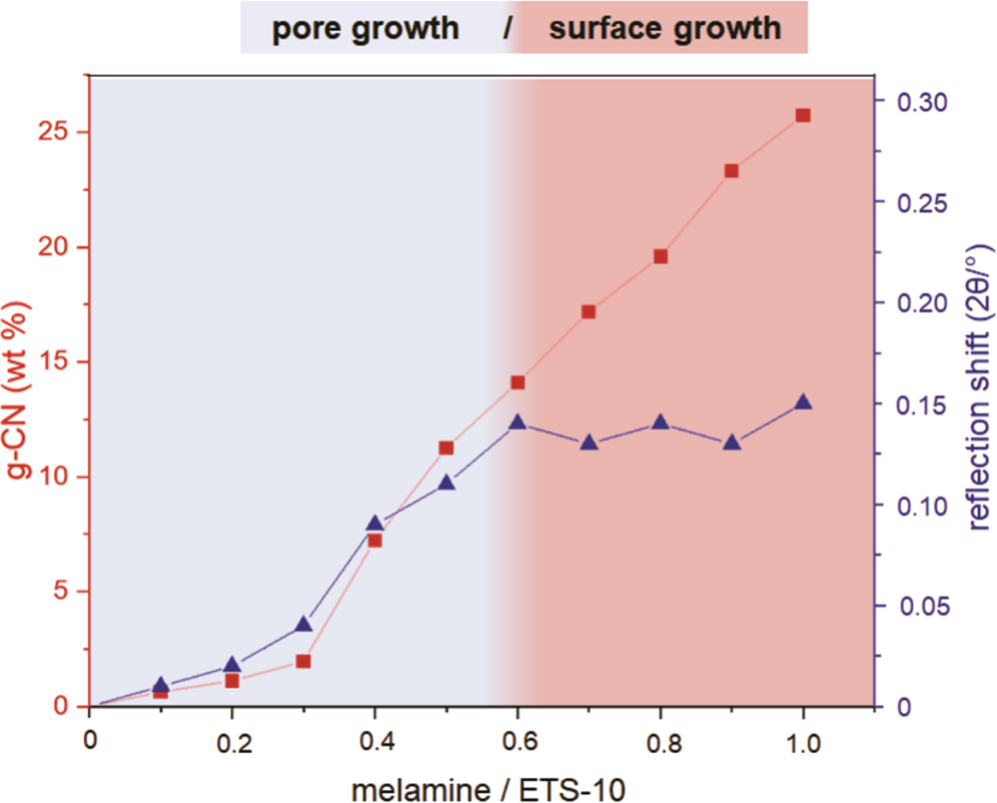
Weight fractions of g-CN and ETS-10 crystal distortion for CN_(0.1–1.0)_/ETS-10 composites prepared with increasing *M*/*T* ratios. The pore (blue) and surface growth (red) of g-CNs are illustrated by coloration.

It can be seen that no structural damage is caused to the ETS-10 framework under the synthesis conditions applied. However, a decrease of the intensities of the first reflections of ETS-10 is observed with CN loading. For zeolites, this is an indication for the filling of the micropores with a guest material. In addition, reflection shifts with increasing CN content were observed for all CN/ETS-10 composites if compared to pristine ETS-10, indicating a progressive distortion of the ETS-10 crystal structure. A constant reflection shift (distortion) for composites with *M*/*T* ratios above 0.6 was observed even though the amount of the carbon nitride material in the composites was constantly growing as illustrated in Figure 2. The distortion of the ETS-10 crystal structure is based on the progressive formation of the carbon nitride material in the microporous system. The fact that at *M*/*T* ratios beyond 0.6, no further distortion occurs indicates that no additional CN is deposited within the micropores. Consequently, *M*/*T* ratios beyond 0.6 must result in the growth of bulk g-CN on the surface of ETS-10 crystallites.

**Figure 3:**
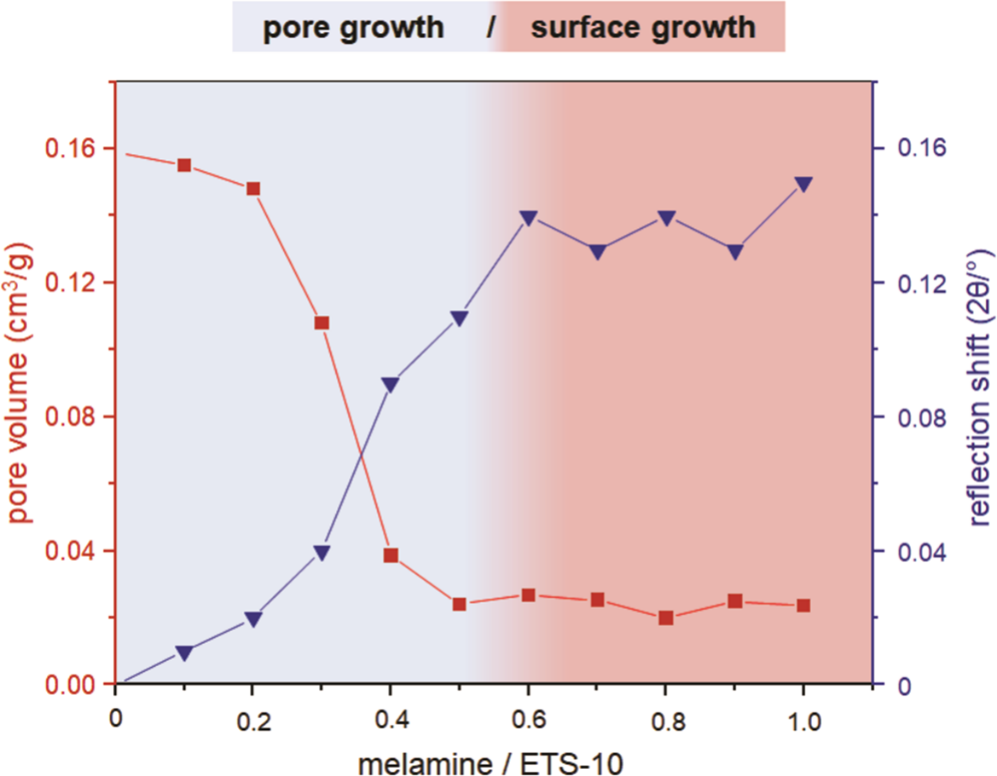
Total pore volume and crystal distortion for CN_(0.1–1.0)_/ETS-10 composites prepared with increasing *M*/*T* ratios. The pore (blue) and surface growth (red) of g-CNs are illustrated by coloration.

**Figure 4:**
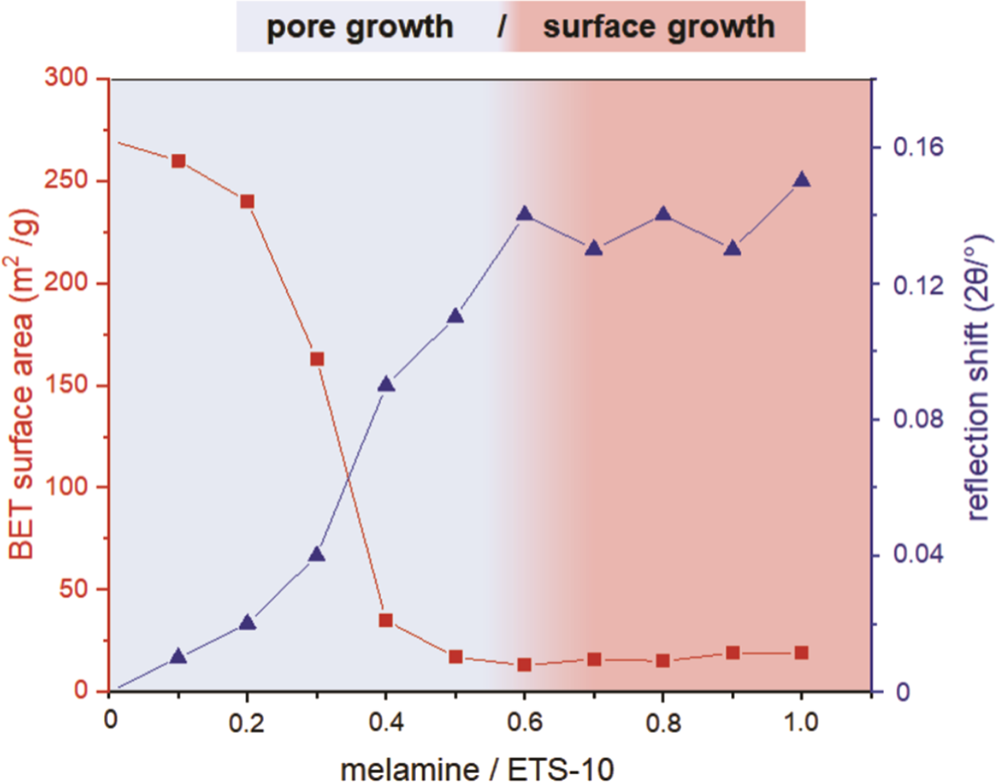
BET surface area and crystal distortion for CN_(0.1–1.0)_/ETS-10 composites prepared with increasing melamine/ETS-10 ratios. The pore (blue) and surface growth (red) of g-CNs are illustrated by coloration.

Decreasing pore volumes and apparent specific (BET) surface areas confirm the progressive pore filling as shown in Figures 3 and 4. The respective N_2_ adsorption–desorption isotherms and pore volume distributions of CN/ETS-10 composites are shown in the Supporting Information (Figures S3–S5). The data show that the distortion in the ETS-10 crystals strongly correlates with the remaining pore volume and BET surface area up to an *M*/*T* ratio of 0.6. A more or less complete loss of micropore volume is observed for all CN/ETS-10 samples synthesized with *M*/*T* ratios higher than 0.6. The remaining pore volume visible in Figure 3 at higher *M*/*T* ratios is that of voids between ETS-10 particles (textural porosity). The observed maximum in ETS-10 crystal distortion correlates with completed micropore filling with a carbon nitride material. The formation of bulk g-CN on the external surface of the ETS-10 crystals is clearly visible by a characteristic yellow coloration of the composites prepared with *M*/*T* ratios above 0.6 (Figure S6).

All changes of the ETS-10 structure (crystal distortion), pore volume, and surface area are completely reversible by heating CN/ETS-10 composites under air at 400 °C for 12 h (Figure S7), indicating that carbon nitride formation took place in and on ETS-10 without any reactions with the ETS-10 template itself.

**Figure 5:**
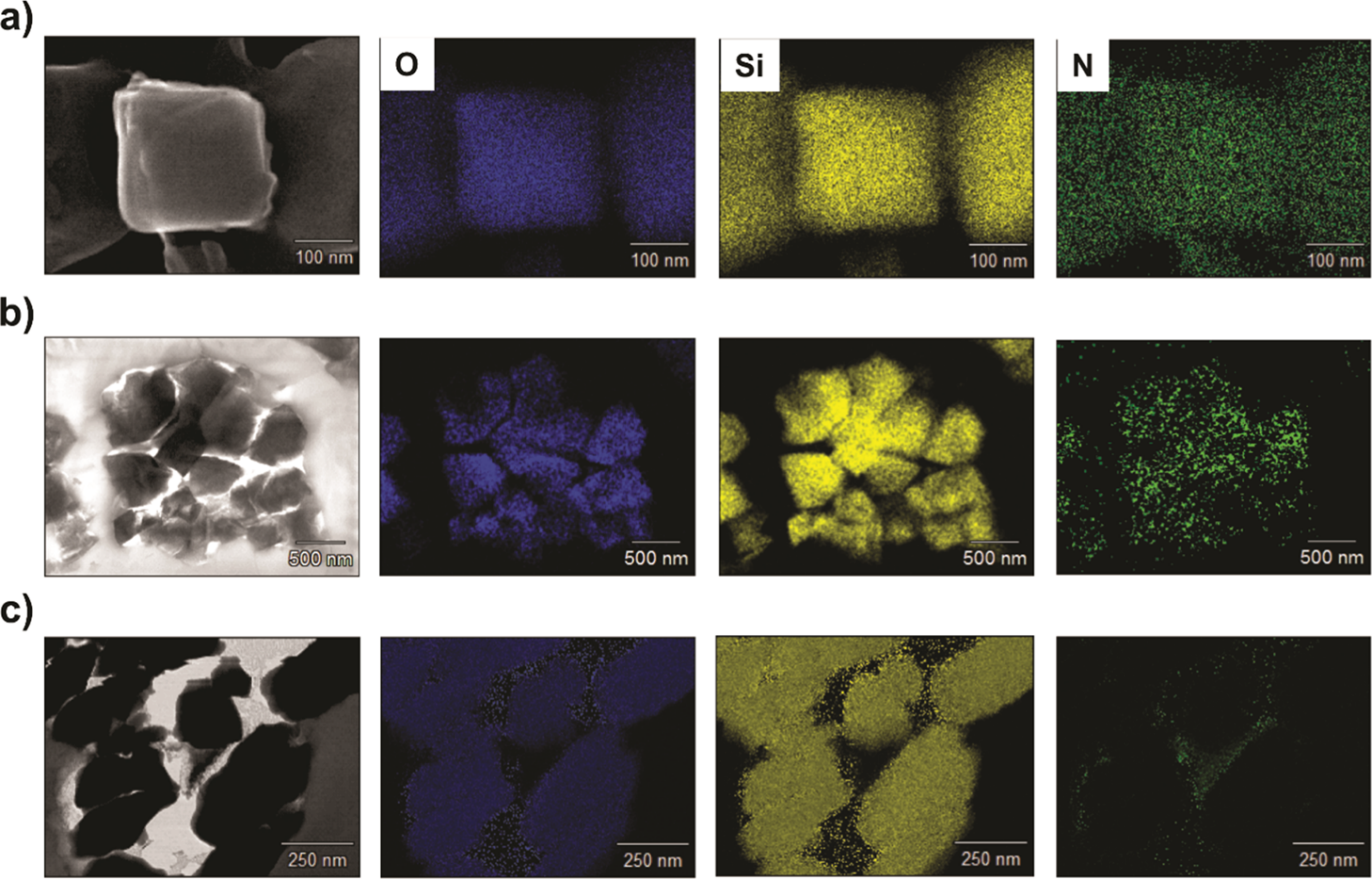
Cross-sectional STEM images of resin-embedded (a) CN_0.5_/ETS-10, (b) CN/Y, and (c) CN/ZSM-5 composites with corresponding EDX mapping for O, Si, and N.

The sterical limit of the g-CN formation in micropores was demonstrated by using ZSM-5 (pore diameter 0.47 nm) and zeolite Y (cage diameter 1.2 nm) as alternative hard templates. Figure 5 shows cross-sectional scanning transmission electron microscopy (STEM)/energy-dispersive X-ray (EDX) images of cuts through CN_0.5_/ETS-10, CN/Y, and CN/ZSM-5 composites embedded in a resin measured either in SEM or in TEM mode. Elemental mapping of CN_0.5_/ETS-10 and CN/Y samples shows a homogenous distribution of nitrogen throughout the silicate crystallites, which are indicated by silicon and oxygen. This again indicates that the micropores of these silicates are homogeneously filled with carbon nitride. These observations are supported by EDX line scans as shown in Figure S9.

While ETS-10 and zeolite Y act as suitable templates for the formation and polymerization of heptazine units, elemental mapping of ZSM-5 samples shows the deposition of nitrogen-containing compounds exclusively on the outer surface of the crystals. The reason for the different carbon nitride depositions on ZSM-5 in comparison to ETS-10 and zeolite Y is likely the fact that the formation of g-CNs within the narrower pores of ZSM-5 (0.47 nm) is impossible for sterical reasons. The molecular size of a heptazine unit, the typical building unit of melon-based carbon nitride, is 0.67 nm, and such a unit does not fit into the narrow pores of ZSM-5. In contrast to that, the pores of ETS-10 and zeolite Y would be large enough to accommodate a heptazine-based carbon nitride polymer. These observations were a first indication that the carbon nitride within the micropores is indeed based on heptazine units.

**Figure 6:**
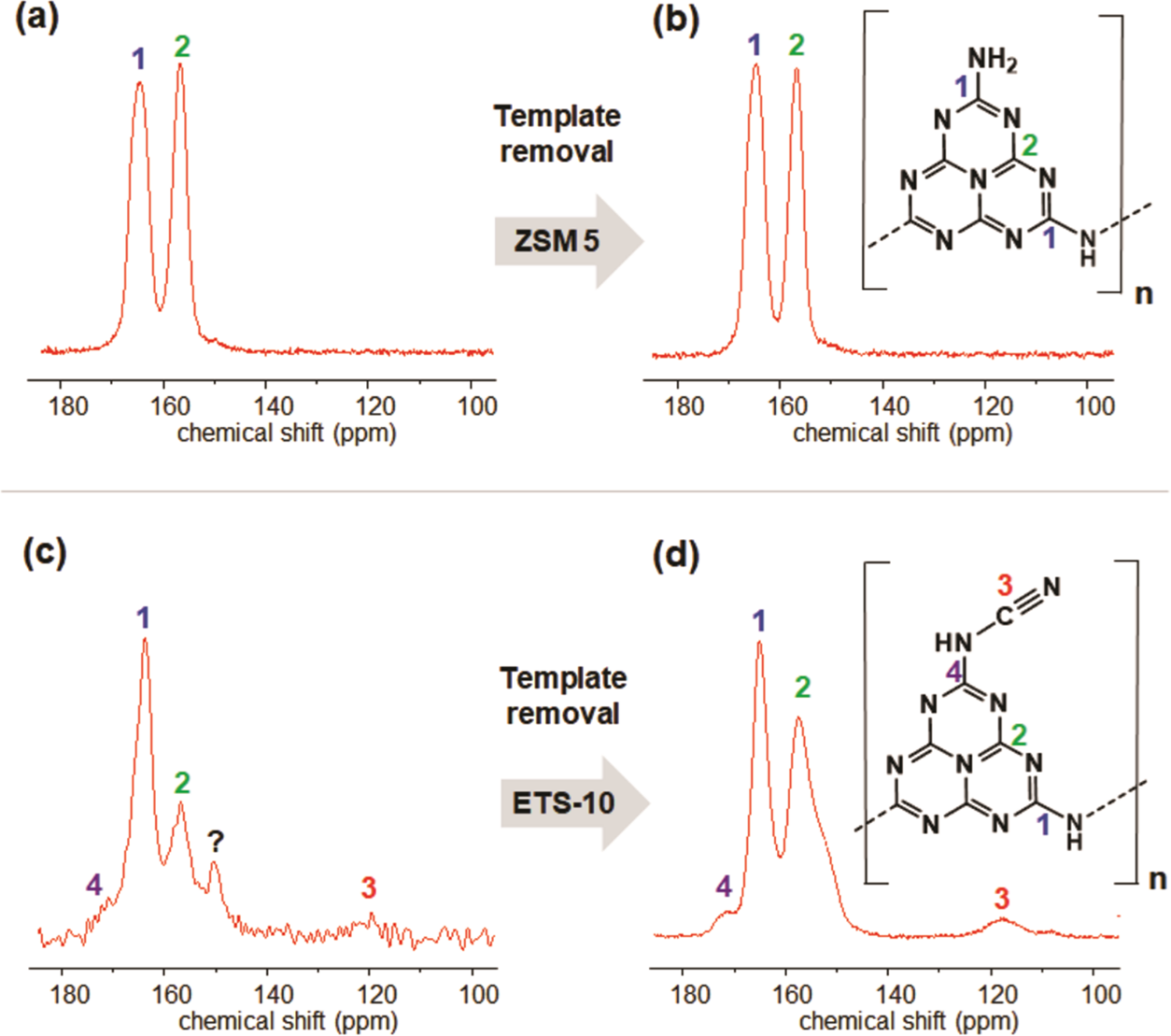
^13^C CP-NMR spectra of (a) CN/ZSM-5 and (c) CN_0.5_/ETS-10 composites and (b) template-free R-CN/ZSM-5 and (d) R-CN_0.5_/ETS-10, respectively.

The structural differentiation of g-CNs before and after template removal was then investigated by solid-state ^13^C cross-polarization NMR spectroscopy (^13^C CP-NMR). The spectra observed for CN/ZSM-5 and R-CN/ZSM-5 (Figure 6a,b) are very similar. This finding is in accordance with the observations of bulk g-CN formation on the surface of ZSM-5 crystals. The lines 1 (165 ppm) and 2 (157 ppm) are characteristic for the sp^2^-hybridized carbon atoms of CN_2_(NH_*x*_) and CN_3_ in heptazine rings of g-CNs.^26^ The spectra of CN_0.5_/ETS-10 and R-CN_0.5_/ETS-10 also show the characteristic lines 1 (163.5 ppm) and 2 (156.4 ppm) before and after template removal. However, a significant increase of the intensity of line 2 is evident for R-CN_0.5_/ETS-10 after template removal. Since the spectra were recorded in cross-polarization mode, the increased intensity of line 2 can be explained by an enhanced transfer of magnetization from additional nearby protons. The formation of intralayer hydrogen bonds between 1D melon strands via primary and secondary amine groups (NH/NH_2_) is the likely reason. This observation supports the hypothesis of a post-synthetic layer formation during the etching process. The lines 3 and 4 observed for CN_0.5_/ETS-10 and R-CN_0.5_/ETS-10 can be attributed to the presence of terminal cyanamide groups as reported by Lotsch et al., who introduced this structural feature via a post-synthetic treatment of bulk g-CN with the aim of increasing the photocatalytic activity.^27^ In our case, the presence of cyanamide groups likely results from partially incomplete heptazine cyclization due to steric restrictions within the micropores of the template. An additional line at ∼150 ppm is clearly visible in the spectrum of CN_0.5_/ETS-10. Currently, we could only speculate about the origin of that signal. However, it is still present as a shoulder next to line 2 in the spectrum of R-CN_0.5_/ETS-10. The NMR data provide evidence that heptazine units are formed within the micropores of ETS-10. Dissolution of ETS-10 then results in a carbon nitride that has some similarity to bulk g-CN but also shows characteristics of a less condensed structure.

The spectra observed for CN/Y and R-CN/Y and those of CN/ZSM-5 and R-CN/ZSM-5 are very similar (Figures S11 and S12). They indicate the absence of terminal cyanamide groups in these materials. However, cross-sectional STEM/EDX images proved the presence of nitrogen species inside the pore system. The large cages of zeolite Y (1.2 nm) apparently allow for complete heptazine cyclization.

**Figure 7:**
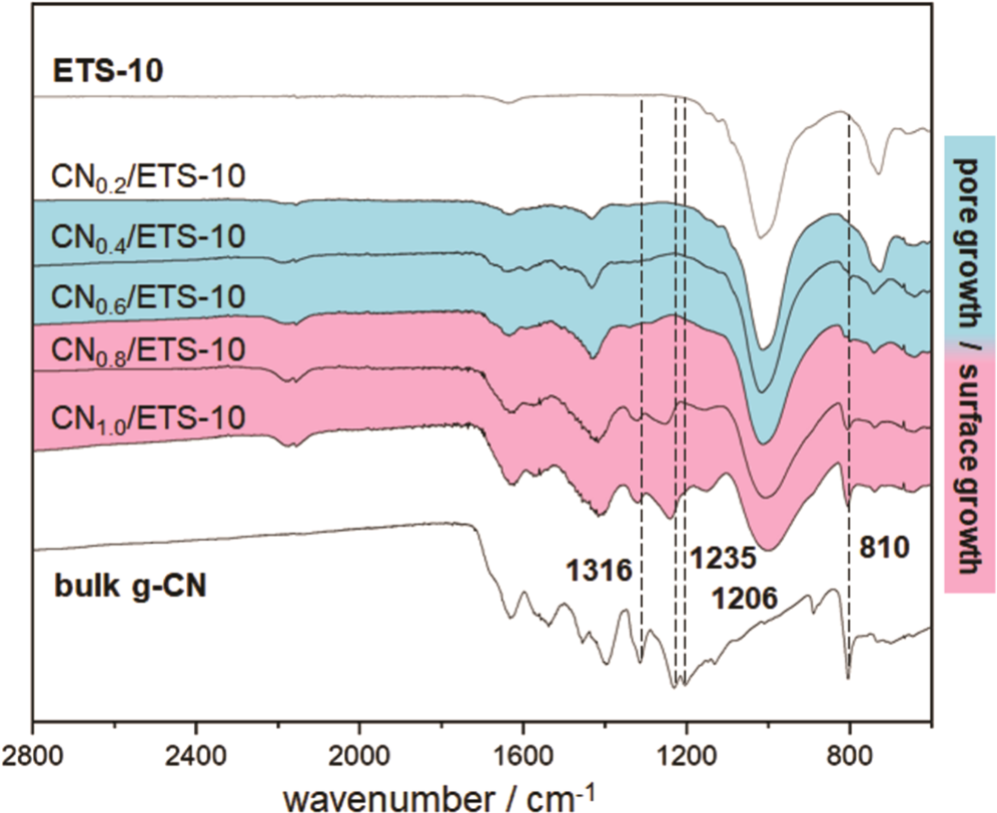
ATR–IR spectra of pristine ETS-10, bulk g-CN, and CN_(0.2–1.0_/ETS-10 composites prepared with increasing melamine/ETS-10 ratios. The pore (blue) and surface growth (red) of g-CNs are illustrated by coloration.

Figure 7 shows attenuated total reflection–infrared (ATR–IR) spectra of pristine microporous ETS-10, bulk g-CN, and CN/ETS-10 composites prepared with increasing *M*/*T* ratios (0.2, 0.4, 0.6, 0.8, and 1.0). The IR spectra of bulk g-CN show prominent absorption bands often described in the literature.^28^ The bands at about 1316, 1235, and 1206 cm^–1^ have been shown to be characteristic for secondary bridging amines between heptazine units.^29^ The presence of this band indicates that the heptazine units are interconnected in the g-CNs on the external surface of the template.

The sharp band at 810 cm^–1^ can be attributed to the breathing mode of the heptazine ring system.^26^ Comparing the spectra of bulk g-CN and CN/ETS-10 composites, these characteristic vibrational bands were exclusively observed for composites containing non-templated carbon nitride material on the ETS-10 crystal surface (melamine/ETS-10 ratio >0.6). Apparently, the vibrational modes of 1D melon strands within the micropores are sterically hindered or suppressed by the confined space in the micropores of ETS-10. These findings are in good accordance with the growth characteristics of g-CNs presented in Figures 2–4, also indicating a surface growth of g-CNs for samples prepared with melamine/ETS-10 ratios above 0.6.

**Figure 8:**
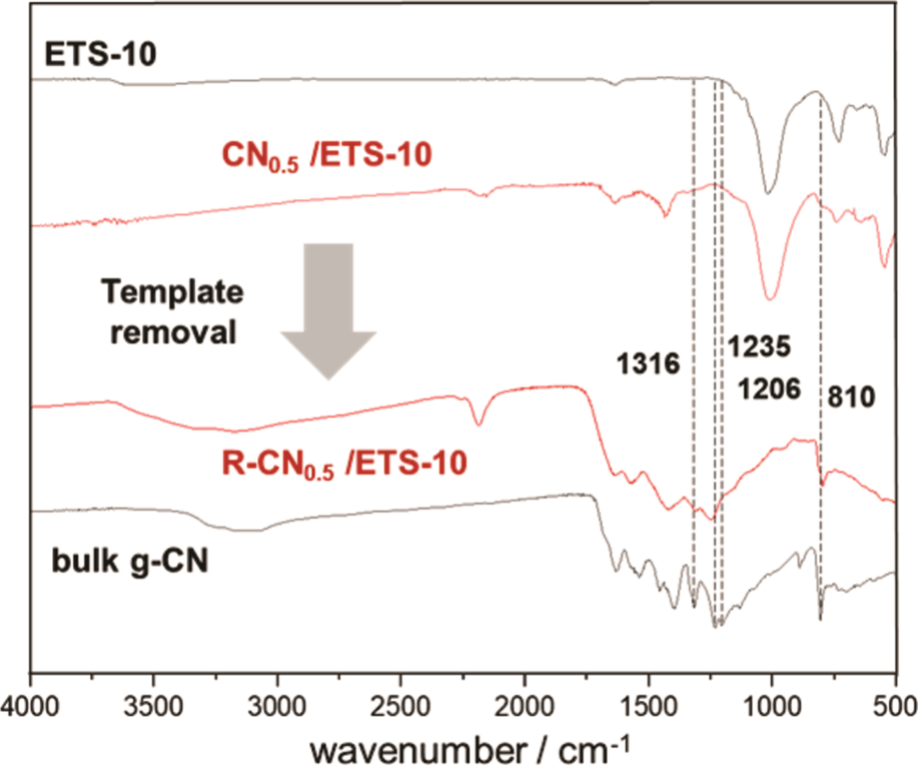
ATR–IR spectra of pristine ETS-10, bulk g-CN, the CN_0.5_/ETS-10 composite, and R-CN_0.5_/ETS-10 after template removal.

The ATR–IR spectra of CN_0.5_/ETS-10 and R-CN_0.5_/ETS-10 in Figure 8 confirm the suppressing influence of the microporous template on vibrational modes of 1D melon strands. While intensities of characteristic absorption bands for R-CN_0.5_/ETS-10 are in good accordance with bulk g-CN, these vibrations are completely or partially suppressed in the CN_0.5_/ETS-10. The absence of absorption bands of bridging amines (1316, 1235, and 1206 cm^–1^) in templated 1D melon samples does not indicate the absence of polymeric species though.^29^ The interconnected units must have existed already in the ETS-10 pores, likely as 1D strands. However, one cannot completely rule out partial intraplanar cross-linking of the respective strands. The vibrational band at about 2185 cm^–1^ can be assigned to C≡N stretching vibrations, confirming the presence of terminal cyanamide groups as indicated by ^13^C CP-NMR.

**Figure 9:**
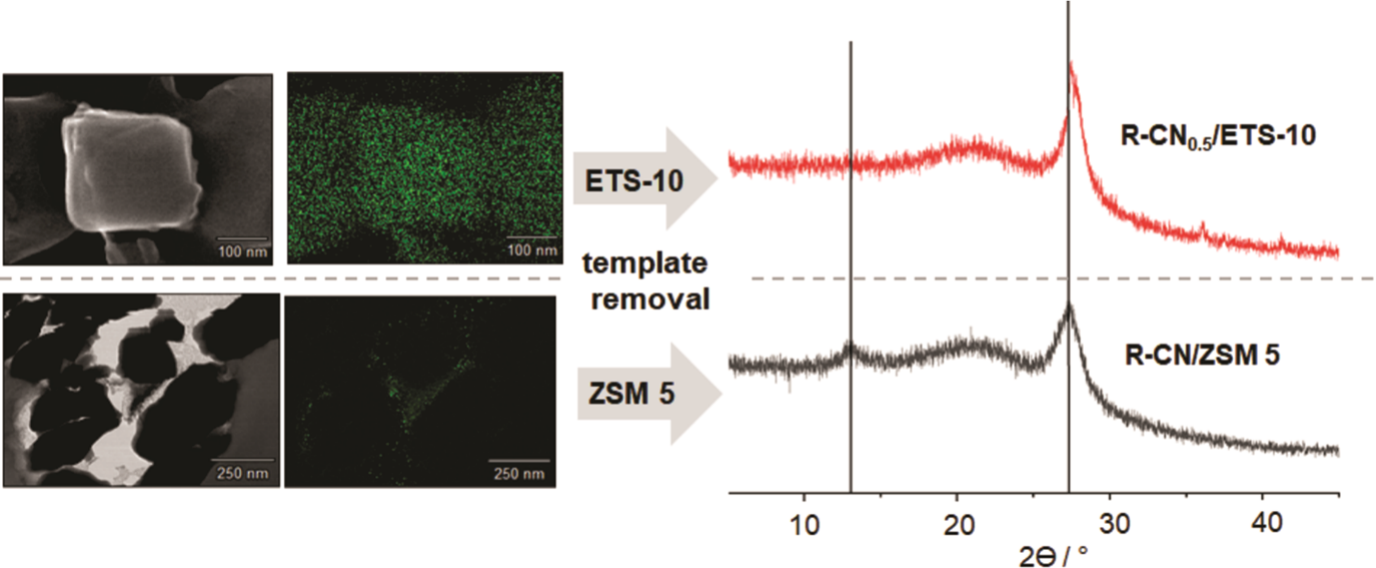
STEM/EDX images of CN_0.5_/ETS-10 and of CN/ZSM-5 and the respective XRD patterns of R-CN_0.5_/ETS-10 and R-CN/ZSM-5 after template removal.

Bulk g-CN obtained by template-free thermal condensation of melamine forms a stacked layered structure and in-plane periodicity.^2^ A typical XRD pattern of g-CNs shows two main reflections: one at ∼27.5° is assigned to the 0.326 nm distance between the stacked g-CN sheets and another at ∼12.8° is assigned to a periodic intralayer d-spacing of 0.688 nm between the melon strands.^30,31^ After removal of the zeolite template, these two reflections are clearly visible in the XRD pattern of R-CN/ZSM-5 (Figure 9), proving the presence of bulk g-CN. In contrast, the template-free R-CN_0.5_/ETS-10 exhibits no in-plane periodicity as no reflection at about 12.8° is visible. Only the reflection at about 28.0° is observed as caused by the layer stacking. Although the formation of layered bulk g-CN on ZSM-5 crystal surfaces is evident, the formation of extended 2D carbon nitride planes within ETS-10 is impeded by the micropore sizes and pore topology. Apparently, a layered 3D structure forms post-synthetically from isolated 1D melon strands during the dissolution of the titanosilicate.

Typical nanocasting routes yield negative replica of pore topologies of the template. In some cases, the negative replica remains in an ordered state, and in some cases, the periodic ordering is lost, for example, if only isolated entities are formed without structure-stabilizing bridges. None of the two scenarios seems to be effective for the carbon nitride formed within the micropores of ETS-10. Instead of forming randomly coiled melon strands, the intrinsic hydrophobic character of melon causes self-assembly based on π-conjugation of heptazine units.^32,33^ In addition to interlayer van der Waals stacking, 2D sheets are stabilized by the formation of intralayer hydrogen bonds between 1D melon chains via primary and secondary amine groups (NH/NH_2_).

## Conclusions

Heptazine-based polymers (melon) can be obtained via a novel 1D to 3D bottom-up route using microporous solids as hard templates. Due to the molecular size of heptazine units (0.67 nm), the sterical restrictions prevent the formation of heptazine-based carbon nitride within hard templates with too narrow micropores as evidenced for the case of ZSM-5. In contrast to that, hard-templating was shown to result in the formation of heptazine within the micropores of materials with large enough pore sizes, such as ETS-10 or zeolite Y. Removal of the hard template via etching was accompanied by the self-assembly of a 1D melon to stacked 3D structures similar but not identical to bulk g-CN. In addition, the confined space in ETS-10 pores allows for the formation of terminal cyanamide groups, which are then also found in the final g-CN materials. Since such groups are known to be active sites for catalytic reactions, the materials reported here may find applications not only in photochemistry but also in other areas of heterogeneous catalysis.

## Supporting Information Available

Analytical methods and complementary characterization of the ETS-10 template and composites, including XRD, TEM images, N_2_ isotherms, and EDX line scans, as well as illustrations of the structures of ETS-10, zeolite Y, and ZSM-5 (PDF)
