# Supplementary material for: From 1D to 3D Graphitic Carbon Nitride (Melon): A Bottom-Up Route via Crystalline Microporous Templates
Source: Inorg Chem. 2021 Dec 2;60(24):18957–63. doi: 10.1021/acs.inorgchem.1c02769 (PMC8693173; doi:10.1021/acs.inorgchem.1c02769)
Supplement: Supplementary file 1 — ic1c02769_si_001.pdf [file ic1c02769_si_001.pdf]

Supporting Information

From 1D to 3D graphitic carbon nitride (melon): A bottom-up route via crystalline microporous templates

Niklas Stegmann[1], Yitao Dai[1], Edward Nürenberg[1], Wolfgang Schmidt[1]*

[1] Max-Planck-Institut für Kohlenforschung, Kaiser-Wilhelm-Platz 1, 45470 Mülheim a.d. Ruhr, Germany

*corresponding author: schmidt@mpi-muelheim.mpg.de

Analytical methods

Powder X-ray diffraction (XRD)

XRD patterns were measured on a Stoe STADI P transmission diffractometer (Debye-Scherrer geometry) and STADI P theta/2theta diffractometer (Bragg-Brentano geometry) geometry using 0.5 mm borosilicate capillaries for transmission measurements. The transmission diffractometer was equipped with a primary germanium monochromator, the reflection instrument was equipped

with an energy-dispersive PIN diode detector. Both instruments were operated with Cu K_{α} radiation.

Scanning electron microscopy (SEM)

SEM micrographs, elementary maps, and EDX line-scans were acquired with a Hitachi H-S5500 electron microscope operated at 30 kV. The samples were measured in resin-embedded sections. The sections were prepared by embedding the sample in a low viscosity resin (*SPURR*) followed by slicing the solid material with a diamond knife (*Mikrotom Reichert-Jung Ultracut*). EDX line-scans consists of 100 points, on which the electron beam remains for 0.1 seconds per point.

Transmission electron microscopy (TEM)

Bright-field TEM images were performed with a *Hitachi H-7100* microscope operated at 100 kV. The samples were measured on a copper TEM grid (400 mesh)

Solid state ^{13}C cross-polarization NMR spectroscopy (^{13}C CP-NMR)

The solid-state ^{13}C NMR spectra were recorded on a Bruker Avance III HD 500WB spectrometer using a double-bearing MAS probe (DVT BL4) at a resonance frequency of 125.8 MHz. The experimental conditions for ^{13}C cross-polarisation (CP) MAS NMR were as follows: 10 kHz spinning rate, 2 s recycle delay, 12,000–144,000 scans, 2 ms contact time, 3.3 μs ^{1}H $\pi/2$ pulse, and high-power proton decoupling (spinal64). The ^{13}C chemical shift was referenced with respect to neat TMS in a separate rotor.

Attenuated total reflection IR (ATR-IR)

ATR-IR spectra were recorded on a PerkinElmer UATR TWO spectrometer equipped with a diamond crystal.

N_2 physisiorption

N_2 physisorption measurements were carried out on a 3Flex sorption instrument from Micromeritics. Samples were degassed on the SmartVacPrep setup from Micromeritics. Specific surface areas (SSA) were calculated with the Brunauer−Emmett−Teller (BET) equation using the MicroActive software from Micromeritics. Total pore volumes were calculated from the volume adsorbed at relative pressures of $p/p_0 = 0.99$.

Complementary characterization

ETS-10 template

ETS-10 was characterized by powder XRD (**Figure S1**), TEM (**Figure S2**), and N_2 physisorption (**Figure S3, S4 and S5**). XRD patterns in **Figure S1** reveal a high crystallinity of the ETS-10 material and a complete consumption of titanium dioxide precursor (P 25) during the hydrothermal synthesis without formation of any by-products. TEM images in **Figure S2** show typical crystal sizes of about 600 nm. Porosity of ETS-10 was indicated by N_2 physisorption revealing an apparent surface area of 260 m^2/g (**Figure S3**).

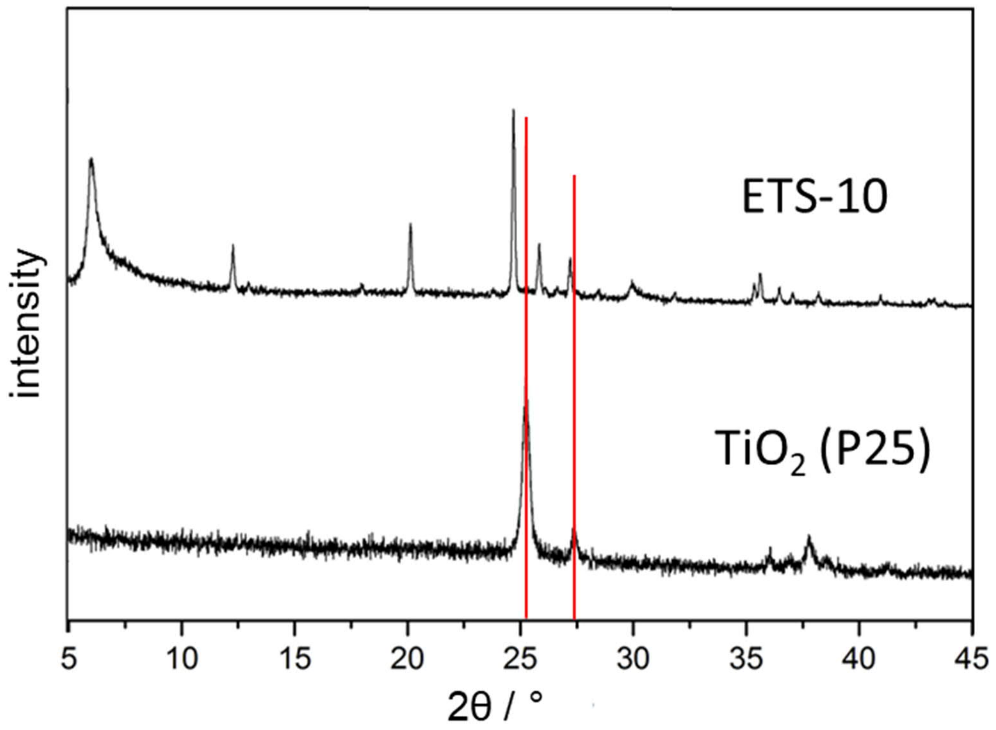

Figure S1. XRD patterns of pristine ETS-10 and TiO_2 (P25). Red lines indicate the main reflections of rutile and anatase phases of the TiO_2 (P25) precursor.

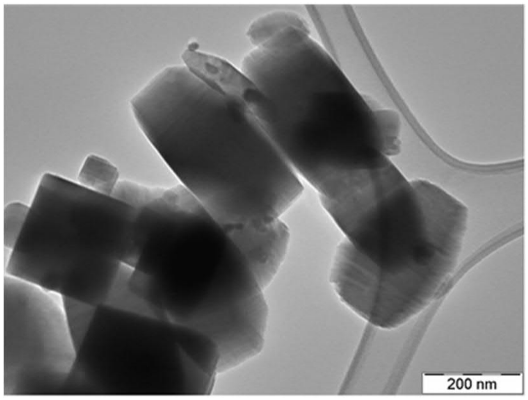

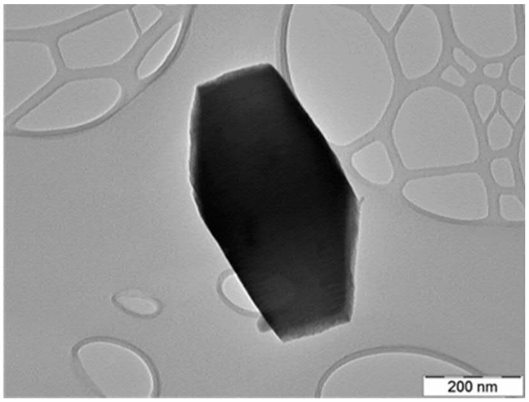

Figure S2. Representative bright-field TEM images of pristine ETS-10.

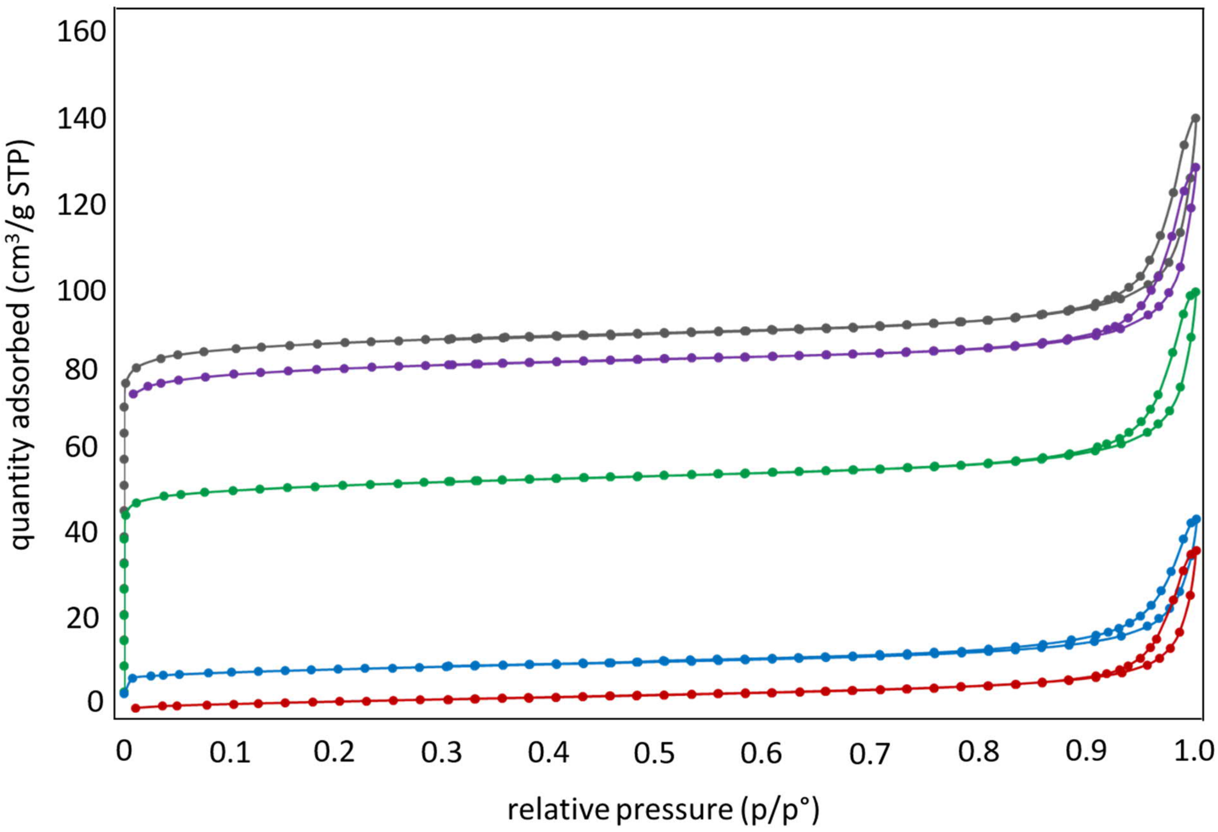

Figure S3. N_2 adsorption-desorption isotherms of pristine ETS10 (▬) and $CN_{0.2-0.6}$/ETS-10 composites (precursor ratio: 0.2 (▬), 0.3 (▬), 0.4 (▬), 0.6 (▬)).

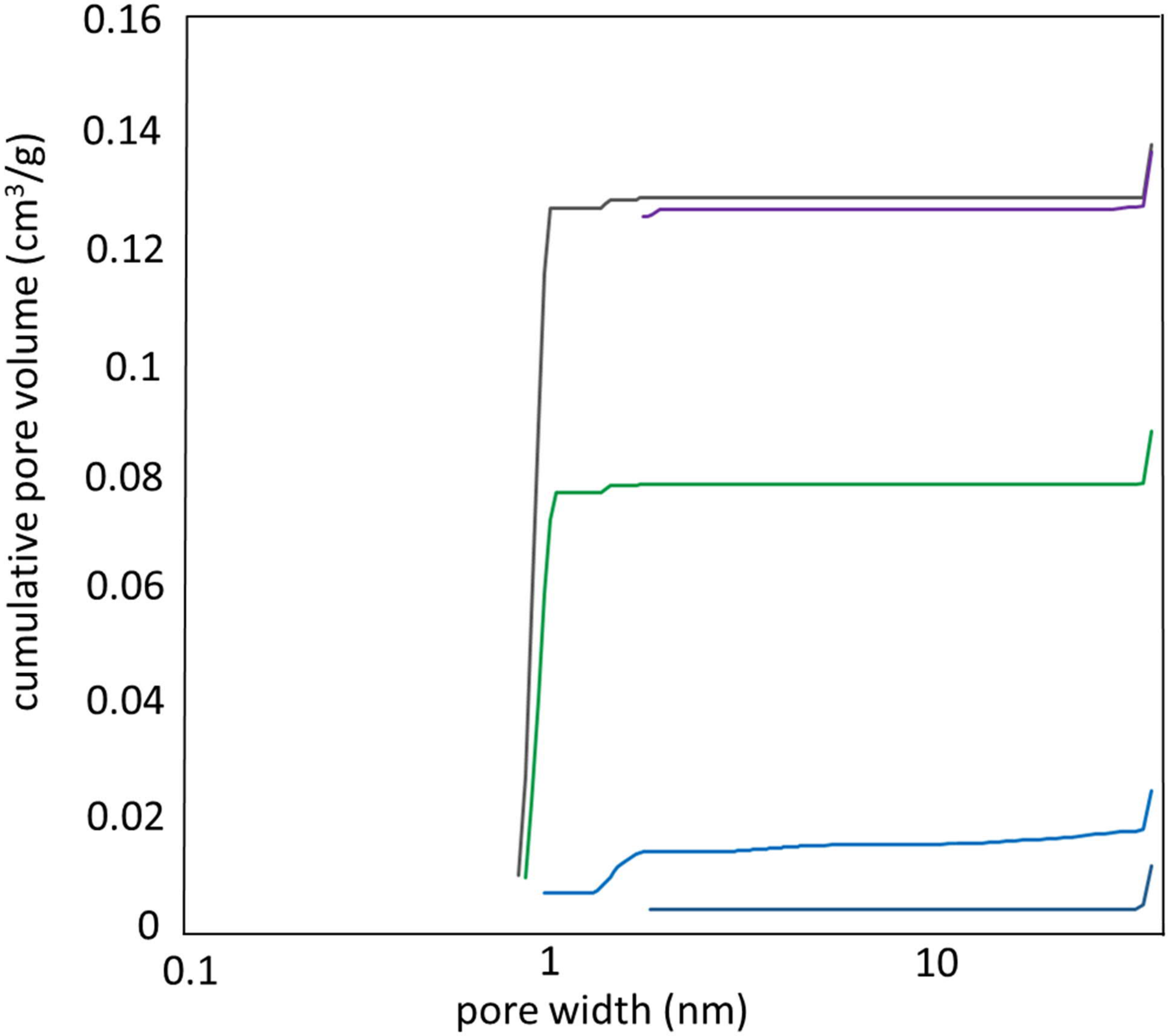

Figure S4: Cumulative pore volume distribution of pristine ETS10 (▬) and $CN_{0.2\text{-}0.6}$/ETS-10 composites (precursor ratio: 0.2 (▬), 0.3 (▬), 0.4 (▬), 0.6 (▬)).

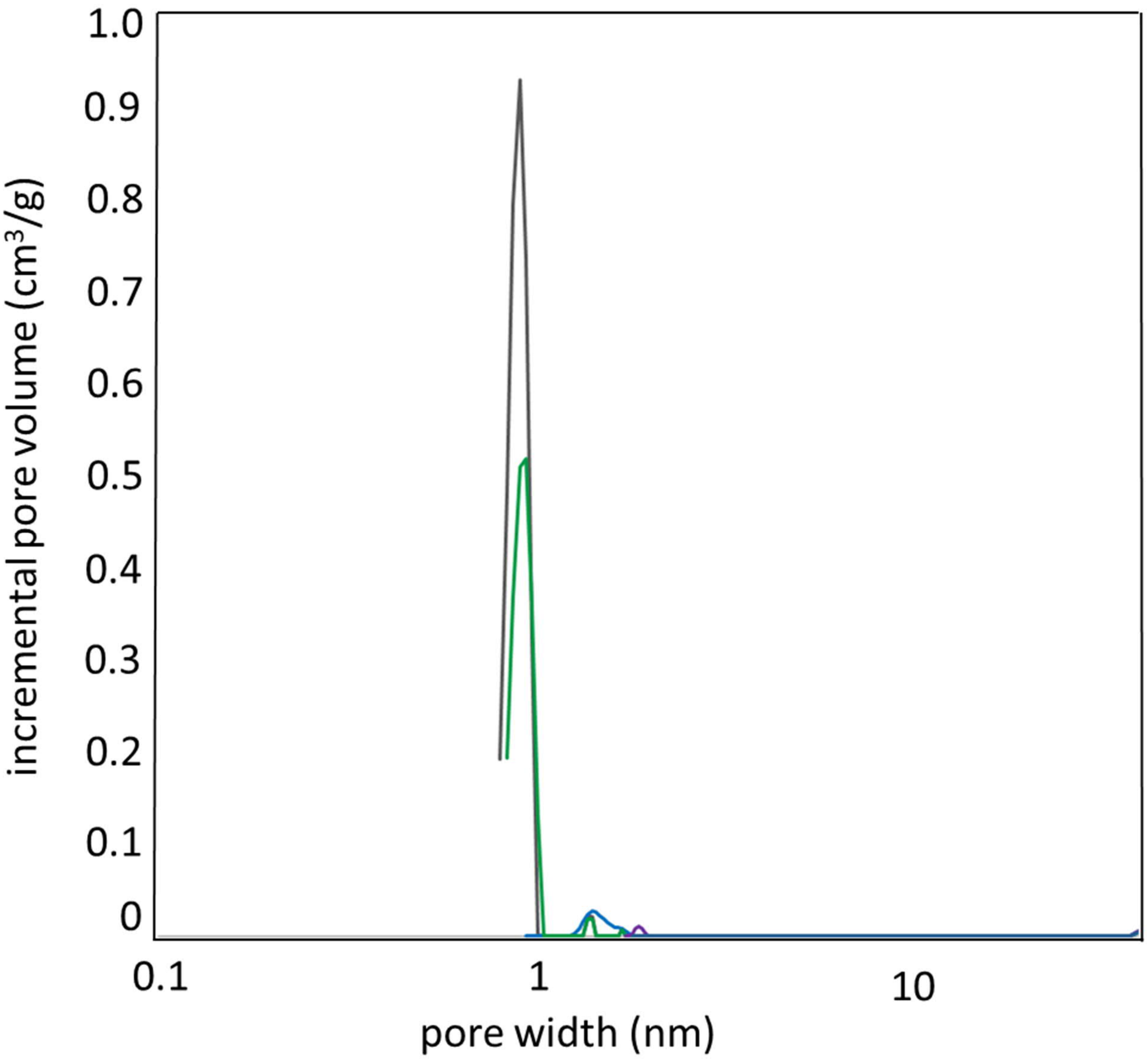

Figure S5. Incremental pore volume distribution of pristine ETS10 (▬) and $CN_{0.2\text{-}0.6}$/ETS-10 composites (precursor ratio: 0.2 (▬), 0.3 (▬), 0.4 (▬), 0.6 (▬)).

CN/ETS-10 composites

Increasing surface growth of bulk g-CN is clearly visible by the characteristic yellow coloration of CN/ETS-10 composites prepared with increasing melamine/ETS-10 ratios (**Figure S6**).

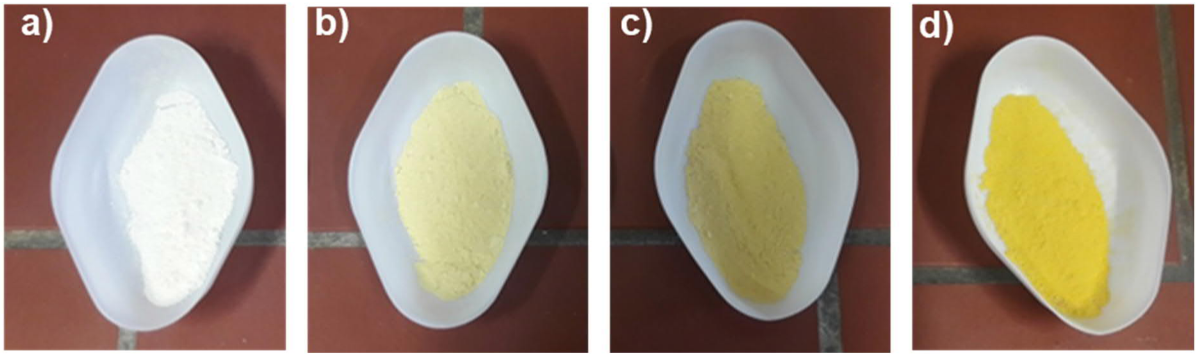

Figure S6. Images of a) $CN_{0.4}$/ETS-10, b) $CN_{0.6}$/ETS-10, c) $CN_{0.8}$/ETS-10, and d) $CN_{1.0}$/ETS-10 composites showing increasing yellowish coloration correlating with increasing surface growth of bulk g-CN.

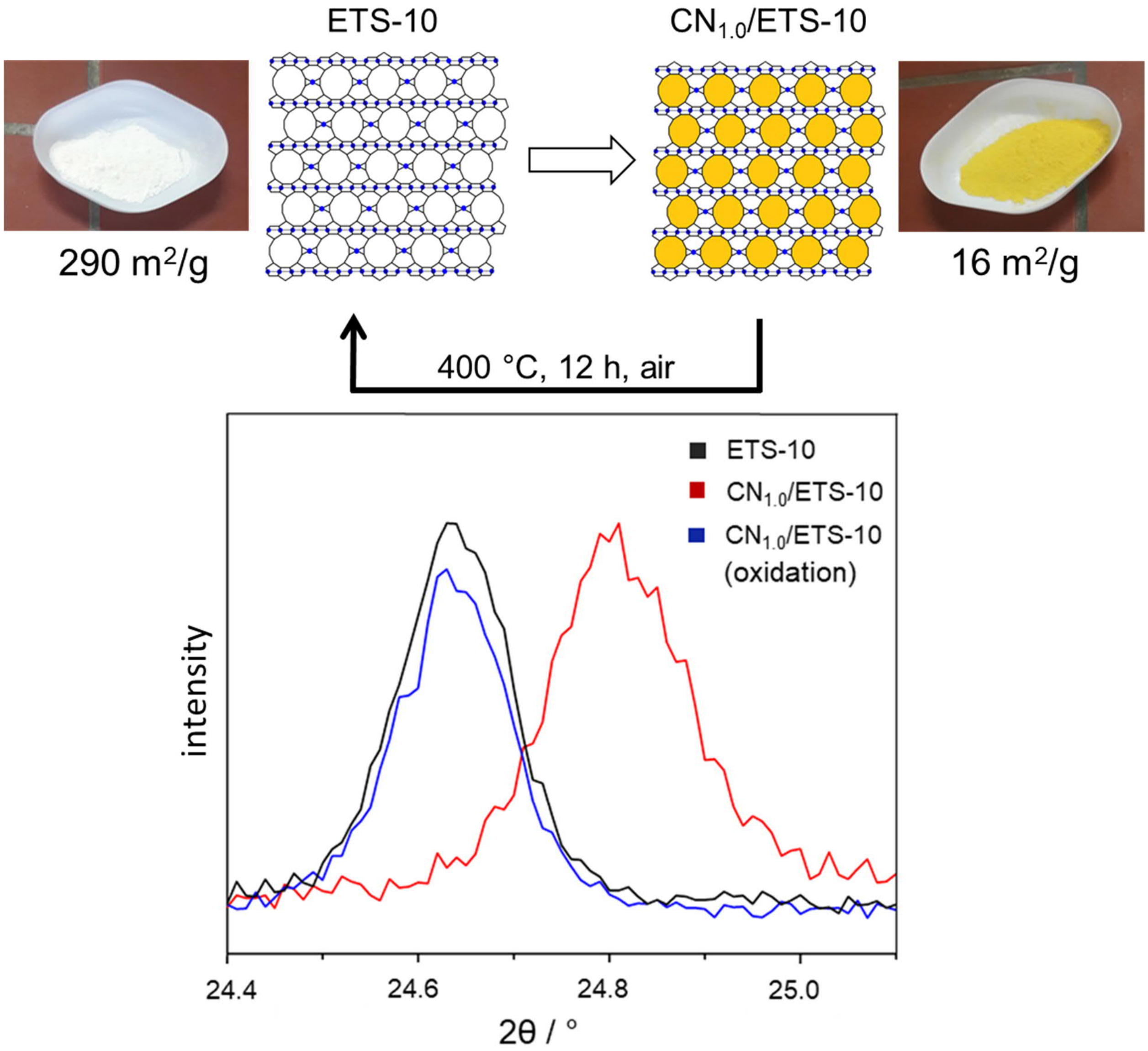

Figure S7. Illustration of reversible pore filling of ETS-10 and emptying by oxidation. All changes in ETS-10 structure regarding crystal distortion, pore volume and surface area are completely reversible by heating $CN_{1.0}$/ETS-10 composite.

As shown in **Figure S8**, both isotherms lie exactly on top of each other, indicating that the CN has been removed completely from the pores of ETS-10.

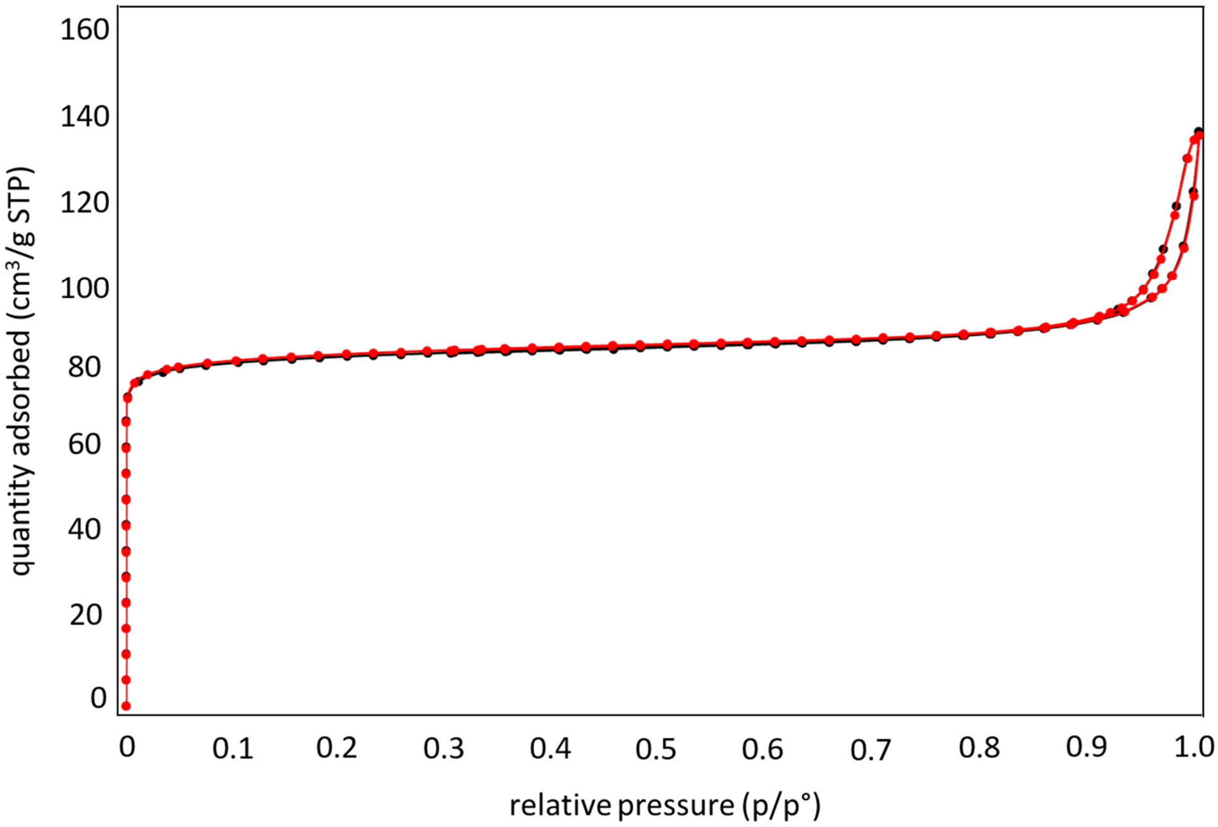

Figure S8. N_2 adsorption-desorption isotherms of pristine ETS-10 (▬) and $CN_{1.0}$/ETS-10 composite after heating for 12 h at 400 °C in air (▬).

Cross-sectional EDX line scans of resin embedded CN/ETS-10 composites in **Figure S9** show a homogenous distribution of nitrogen and carbon atoms inside of ETS-10 template matching the shapes of the crystals as indicted by silicon atoms incorporated in the crystal structure of the framework.

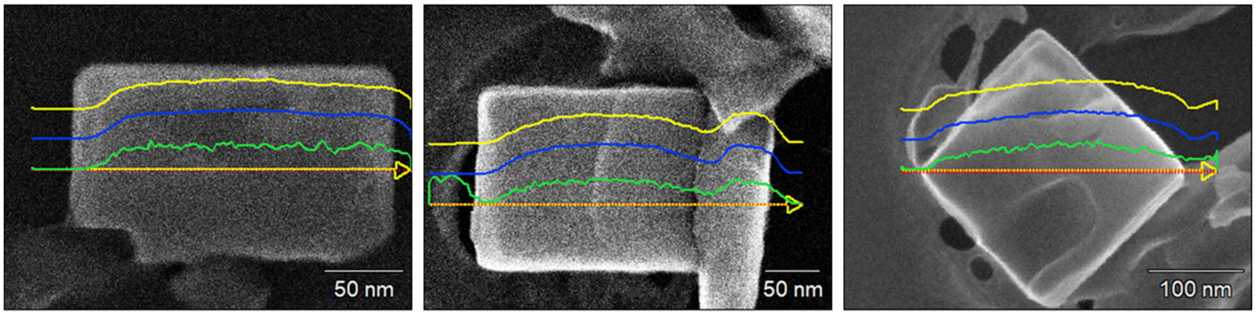

Figure S9. SEM images of $CN_{0.5}$/ETS-10 composite particles with cross-sectional EDX line scans showing N(▬), O(▬) and Si(▬) concentrations along the crystallites.

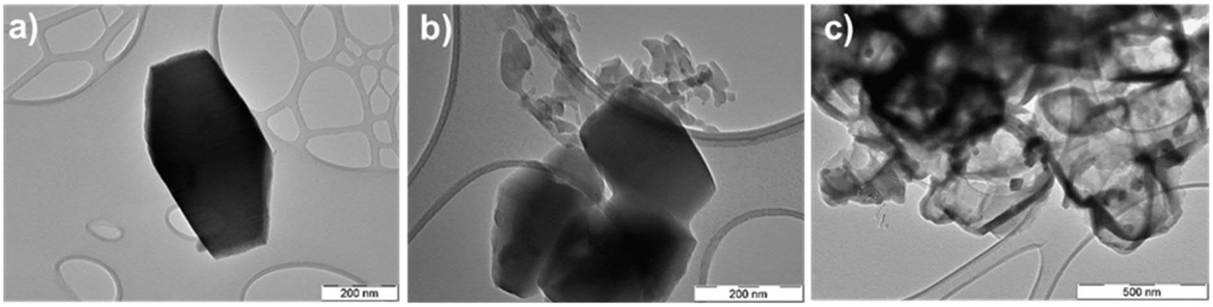

Figure S10. Representative bright-field TEM images of (a) pristine ETS-10, (b) $CN_{1.0}$ /ETS-10 composite and (c) core-shell like R-$CN_{1.0}$/ETS-10.

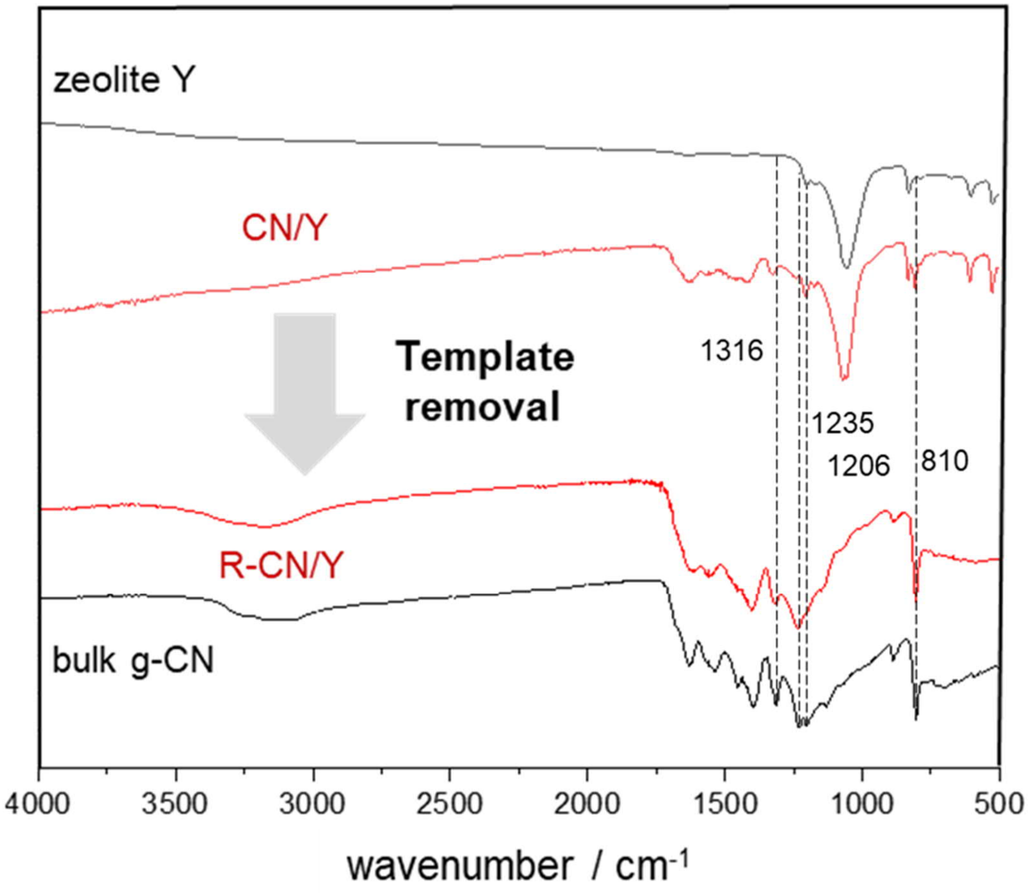

Figure S11. ATR-IR spectra of zeolite Y, bulk g-CN, CN/Y composite and R-CN/Y after template removal.

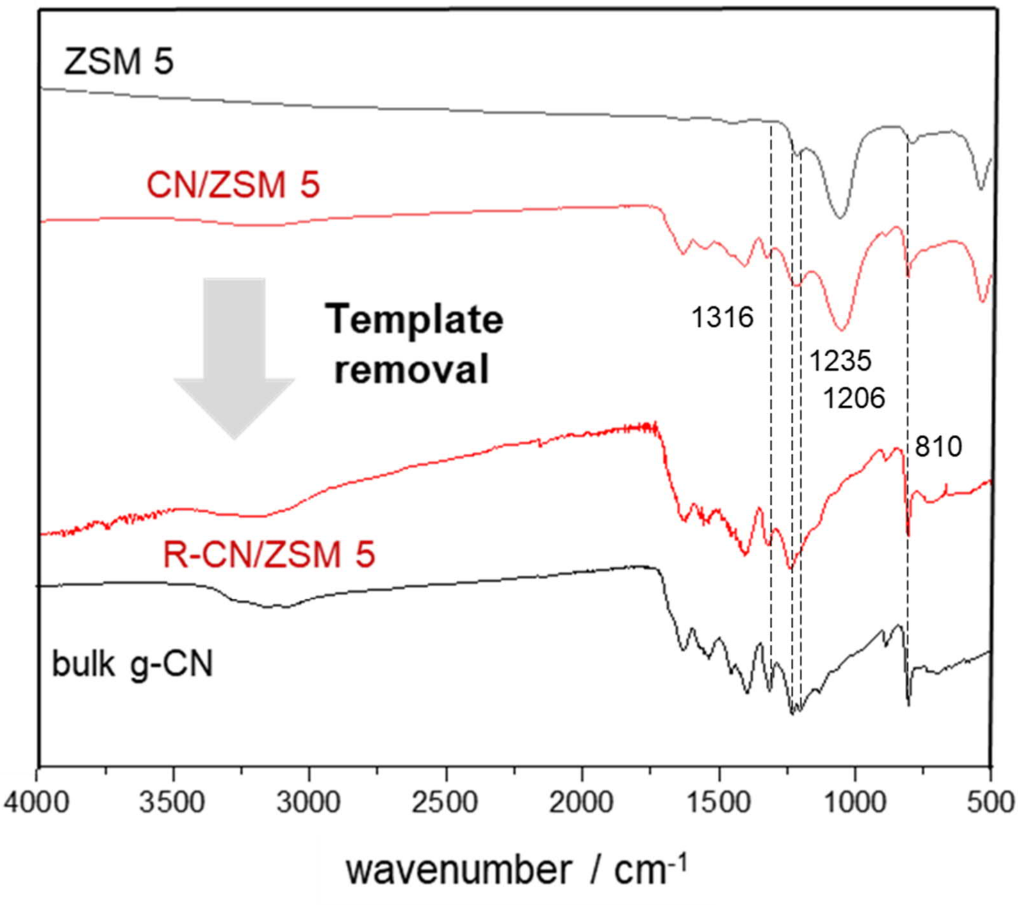

Figure S12. ATR-IR spectra of ZSM 5, bulk g-CN, CN/ZSM 5 composite and R-CN/ZSM 5 after template removal.

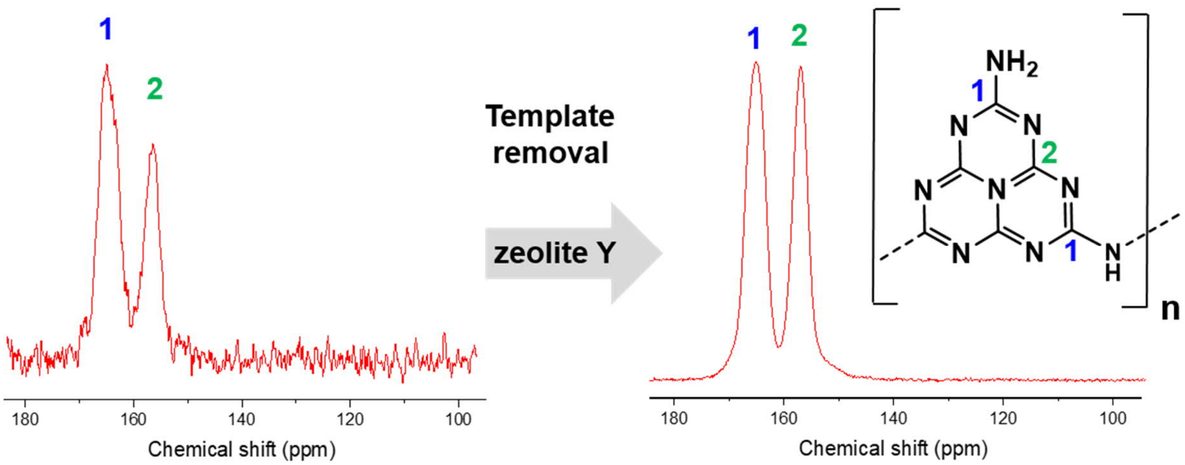

Figure S13. ^{13}C CP-NMR spectra of (left) CN/Y composite and (right) R-CN/Y after template removal.

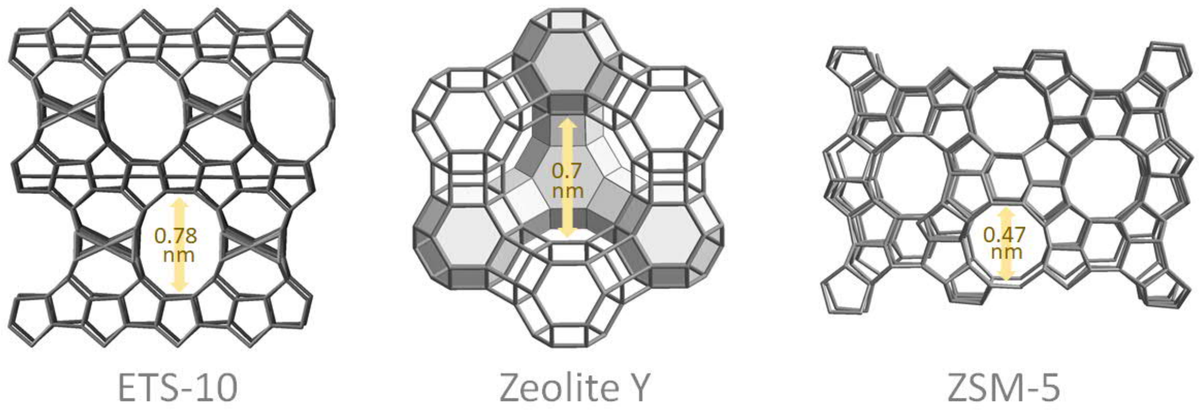

Figure S14. Structure topologies of ETS-10, Zeolite Y, and ZSM-5. Oxygen positions are omitted for better visibility. The numbers within the pore windows indicate the pore diameters.

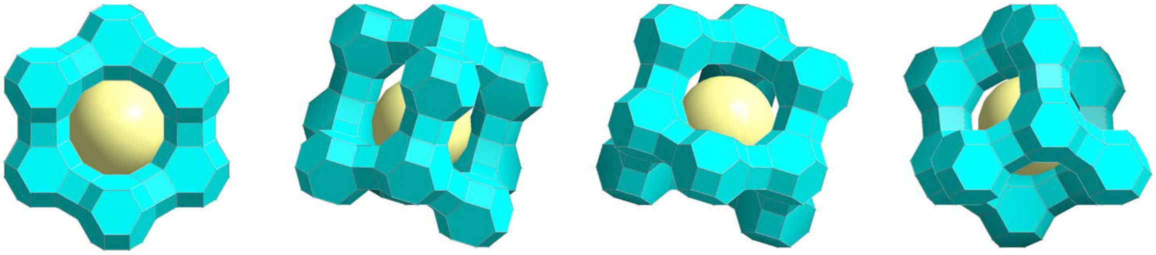

Figure S15. Visualization of the cage size within Zeolite Y. The yellow sphere shown in the cage has a diameter of 1.2 nm.
